# Hyphenated Mass Spectrometry-Based Strategies for Characterizing Oligomers of α‑Synuclein in Parkinson’s Disease

**DOI:** 10.1021/acscentsci.6c00514

**Published:** 2026-08-20

**Authors:** Raya Sadighi, Andrea Istrati, Sigourney Karijodikoro, Guus Scheefhals, Melissa Baerenfaenger, Anouk M. Rijs

**Affiliations:** † Division of BioAnalytical Chemistry, Department of Chemistry and Pharmaceutical Sciences, Amsterdam Institute of Molecular and Life Sciences, 1190 Vrije Universiteit Amsterdam, De Boelelaan 1108, 1081 HZ Amsterdam, The Netherlands; ‡ Centre for Analytical Sciences Amsterdam (CASA), The Netherlands; § Syngle Therapeutics B.V., https://www.syngletherapeutics.com/, The Netherlands

## Abstract

The pathological aggregation of α-synuclein (α-syn), an intrinsically disordered protein that regulates synaptic vesicle trafficking in the brain, is a defining molecular feature in Parkinson’s disease (PD). Early oligomeric assemblies are widely considered the most neurotoxic species, yet their structural features remain unexplored due to their low abundance, transient, and fragile nature. Here, we establish a trapped ion mobility-mass spectrometry (TIMS-MS) strategy that enables direct detection and structural interrogation of these oligomers. Low-flow size exclusion chromatography provides MS-compatible desalting and reveals broad ensemble-level differences between fresh and aggregated α-syn. Native TIMS-MS measurements uniquely probe numerous higher-order oligomers, thereby revealing a reduced conformational heterogeneity with increasing oligomer size, supporting a linear β-strand-type assembly pathway. These findings show that oligomerization drives a transition from dynamic monomers to increasingly ordered intermediates. This progressive rigidification is relevant to models in which α-syn oligomerization alters normal membrane engagement during PD-associated aggregation.

## Graphical abstract

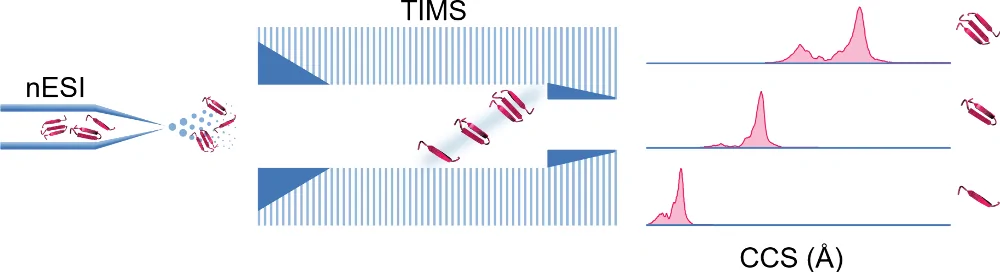

## Introduction

The misfolding and aggregation of proteins into insoluble, β-sheet-rich amyloid fibrils is a well-known hallmark of neurodegenerative disorders.^1^ In Parkinson’s disease (PD), the formation of soluble oligomers from α-synuclein (α-syn) is thought to be a key factor in the progression of the disease.^2–5^ α-Syn is a 140 amino acid protein abundantly expressed in the brain, primarily localized at presynaptic terminals, where it is thought to play a role in synaptic vesicle trafficking and the regulation of neurotransmitter release.^6^ Structurally, the protein is composed of three functional domains: a membrane-binding N-terminal region (residues 1–66), the aggregation-prone nonamyloid-β component (NAC) domain (residues 67–96), and a highly acidic, unstructured C-terminal region (residues 97–140).^7,8^ The NAC region, particularly the NACore segment (residues 68–78), is critical for fibril formation and has been shown to self-assemble into cross-β structures.^9–12^ The exact mechanism of aggregation is not fully understood, however, increasing evidence implicates small, soluble, oligomeric intermediates of α-syn, rather than mature fibrils, as the most neurotoxic species.^13,14^ These oligomers can interact with lipid membranes, disrupt cellular homeostasis, and contribute significantly to disease progression.^15,16^ For instance, the amphipathic N-terminal helices facilitate membrane binding, potentially promoting toxicity, while the C-terminal domain modulates calcium binding and protein–protein interactions.^17–19^ α-Syn is categorized as an intrinsically disordered protein (IDP), lacking a fixed three-dimensional structure and existing instead as an ensemble of dynamic conformations.^20^ Although biologically functional, this structural plasticity poses significant challenges for characterizing the protein, especially during early aggregation events.

Depending on the stage of aggregation, different analytical techniques are employed to identify and characterize α-syn species, including monomers, oligomers, and fibrils. Mature amyloid fibrils can be structurally resolved using high-resolution techniques such as electron microscopy (EM),^21–23^ X-ray diffraction,^24,25^ and solid-state nuclear magnetic resonance (ssNMR).^26–28^ Aggregation kinetics are commonly monitored using fluorescence-based assays, such as thioflavin T (ThT), which fluoresces upon binding to β-sheet-rich structures. However, ThT fluorescence is only detectable once significant β-sheet content has formed, making it more suitable for tracking later stages of aggregation rather than early oligomer formation.^29–31^

Native mass spectrometry (nMS) has emerged as a powerful technique for detecting early stage α-syn oligomers under near-physiological conditions.^32,33^ Yet, the inherent heterogeneity and structural complexity of these species often result in overlapping mass-to-charge (*m*/*z*) signals or conformational isomers that cannot be distinguished by MS alone.^34^ To resolve this complexity, a separation step is typically required. This can be achieved either prior to MS analysis, by coupling size exclusion chromatography (SEC) to MS, or within the mass spectrometer itself using ion mobility mass spectrometry (IM-MS). SEC is a widely used technique for separating proteins based on their hydrodynamic volume.^35^ However, its application to IDPs such as α-syn is challenging due to their extended and dynamic conformations, which often result in elution behavior that does not correlate with the expected molecular weight.^36,37^ To overcome these limitations, SEC has been coupled with techniques such as dynamic light scattering (DLS) and multiangle laser light scattering (MALLS) to characterize large α-syn protofibrils in the high molecular weight range.^38,39^ SEC coupled to MS is commonly used for the separation and online desalting of stable protein aggregates and has proven effective in biopharmaceutical analysis.^40^ However, despite its growing utility in native protein characterization, the advantage of employing SEC-MS for IDPs remains limited, especially for probing low-abundant and transient α-syn oligomers.

MS in combination with ion mobility (IM) spectrometry adds an additional dimension by separating ions based on their size, shape, and charge, providing an averaged collision cross section (CCS) which reflects the overall shape and conformation of ions in the gas phase.^41^ IM-MS, particularly in the form of traveling wave ion mobility (TWIMS) and drift tube ion mobility (DTIMS) has been applied in the characterization of α-syn. These studies primarily focused on how various factors, such as solution conditions,^42,43^ oxidation,^44,45^ interactions with detergents and lipid vesicles,^46–48^ metal ions,^49–52^ and small molecules^47,53–55^ affect the overall structure and stability of the α-syn monomer. A few studies focused on exploring the conformational landscape of α-syn monomer,^49,56,57^ revealing that it exists in a dynamic ensemble characterized by broad charge state distributions (typically ranging from 6+ to 18+) and wide CCS ranges, reflecting both compact and extended conformations.

While TWIMS and DTMS have been valuable in revealing structural insights into the monomeric and dimeric forms of α-syn, their application to higher-order oligomers is limited, with only one study reporting up to a hexamer.^58^ This is largely due to their reliance on extended drift paths and high electric fields to achieve resolution, which can induce ion activation and collisional heating, often leading to the dissociation of fragile oligomeric species.^41^ Trapped ion mobility spectrometry (TIMS) provides an alternative approach with high mobility resolution and sensitivity, while allowing extensive control over experimental parameters. This facilitates gentler operating conditions, improving the ability to preserve and resolve low-abundant, transient α-syn oligomers.^59^ As a result, TIMS is well-suited to study early stage α-syn oligomers. Nevertheless, the use of TIMS to probe α-syn aggregation remains largely unexplored, with current studies mainly examining the conformational heterogeneity of the monomer, including its modified proteoforms such as O-GlcNAc-modified α-syn.^60^

In this study, we establish a multimethod analytical platform to characterize the structure of early stage α-syn oligomers. Because SEC and TIMS remain underutilized for intrinsically disordered proteins such as α-syn, we evaluate the capabilities of these methods individually and in combination to probe the conformational and oligomeric landscape of α-syn. Therefore, a low-flow SEC-TIMS-MS workflow is employed to resolve compact and extended monomer conformations. The TIMS-MS platform is subsequently advanced by integrating a soft direct-infusion strategy using online nanoelectrospray ionization (nanoESI) to detect higher-order oligomeric species. Across both configurations, TIMS parameters are systematically optimized to enable structural characterization of α-syn oligomers. By combining oligomer detection with structural interrogation, this work defines the conformational transitions accompanying early α-syn assembly and places these changes in the context of Parkinson’s disease.

## Experimental Section

Full experimental details, including materials, instrumentation, and procedures are provided in the Supporting Information (Section S1 and Table S1).

## Results and Discussion

### SEC-TIMS-MS for Characterization of α-syn Monomer

SEC is a widely used technique for separating proteins and their stable aggregates under native conditions.^61,62^ In this study, SEC was employed to explore its potential for separating and analyzing early oligomers of α-syn, or at least to resolve monomers from higher-order species. A low-flow SEC column was used because its low flow rate (16 μL/min) enhances ionization efficiency, particularly for large proteins. It also minimizes shear forces during separation, thereby significantly reducing the risk of protein unfolding prior to MS analysis.^63^ Figure 1A presents the SEC-UV chromatogram of freshly prepared α-syn, resulting in a single peak with a retention time (RT) of 10.4 min. Using a standard protein mix (bovine serum albumin (BSA, 66.5 kDa), cytochrome C (12.3 kDa), and uracil (112 Da)) to calibrate, the α-syn peak exhibited an apparent molecular weight comparable to that of BSA, despite its monomeric molecular weight being approximately 14.5 kDa. The hyphenation of SEC with MS revealed that the peak consists mainly of α-syn monomer, in both a folded conformation with a charge state distribution (CSD) centered around the 8+ monomer ([1]^8+^), and an unfolded structure with a CSD centered around [1]^17+^ (see Figure 1B). Additionally, a low abundance of dimeric species was observed centered around a CSD of 13+ dimer ([2]^13+^). The early elution of α-syn is likely attributed to its intrinsically disordered nature, which gives rise to an expanded hydrodynamic radius, causing it to behave like a larger globular protein during SEC. This behavior is consistent with prior studies reporting apparent molecular weights for monomeric α-syn in the range of 44–55 kDa.^64^

**Figure 1:**
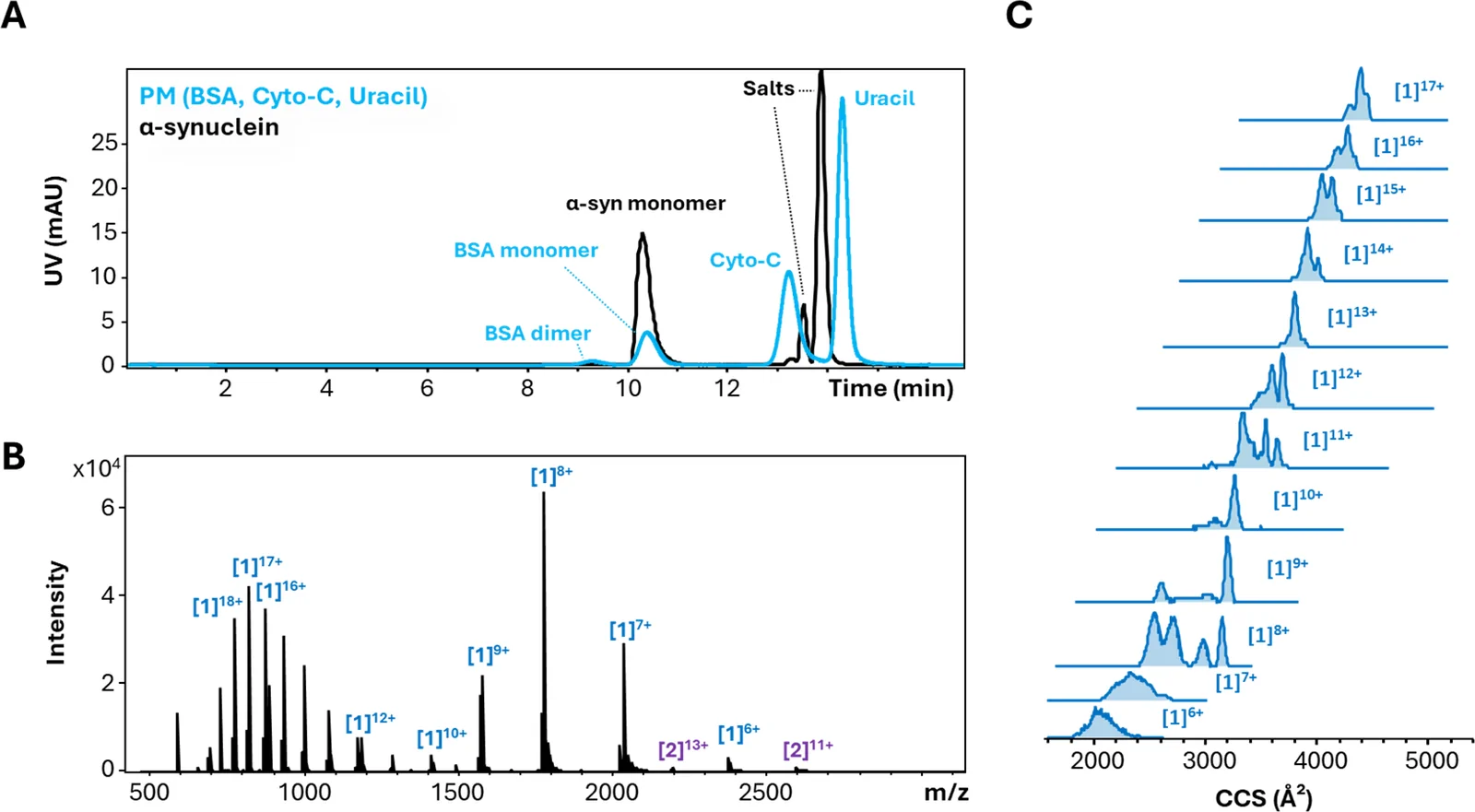
SEC-UV-TIMS-MS of fresh α-syn. (A) SEC-UV chromatogram of fresh α-syn (black) at a flow rate of 16 μL/min overlaid on a protein mix showing similar elution time behavior to BSA. (B) SEC-MS of fresh α-syn extracted between min 10 to 11, resulting in two distinct charge state distributions. (C) SEC-TIMS of fresh α-syn where ^TIMS^CCS_N2_ values are plotted for all charge states of the monomer.

Figure 1C presents the conformational landscape of monomeric α-syn as derived from TIMS-based CCS distributions (^TIMS^CCS_N2_). TIMS fingerprints are shown for charge states ranging from [1]^6+^ to [1]^17+^ with all instrumental parameters optimized for monomer transmission (Table S1). For the lower charge states ([1]^6+^ and [1]^7+^), α-syn predominantly populates compact conformations, with ^TIMS^CCS_N2_ values in the range of approximately 1500–2500 Å^2^. These compact states are characterized by broad, unresolved mobility features rather than discrete ^TIMS^CCS_N2_ peaks. Such peak broadening reflects pronounced conformational heterogeneity within the monomeric ensemble and is consistent with the presence of multiple compact conformers with similar CCS values. Because the TIMS method was set to cover the full α-syn conformational range, using a broad mobility window and relatively short ramp time, the compact low-charge states were not fully resolved. As a result, these conformers appear as an averaged broad ^TIMS^CCS_N2_ distribution.

[1]^8+^ represents the most abundant charge state within the monomeric α-syn CSD. Its ^TIMS^CCS_N2_ profile exhibits multiple, partially resolved conformational families spanning both compact and extended regions of the monomer. This behavior is consistent with previous ion mobility studies, which similarly report that monomeric α-syn populates both compact and extended conformations in intermediate charge states, with [1]^8+^ displaying several distinct conformational families within a ^TIMS^CCS_N2_ range of approximately 2200–3000 Å^2^.^49^

At 1808 *m*/*z*, the most extended conformation observed at approximately 3000 Å^2^ is attributed to the extended monomeric state of α-syn. A complete overview of the mobility profiles for all monomeric charge states is provided in Figure S1.

At [1]^9+^ and [1]^10+^, we observe a transitional behavior from folded to unfolded forms, where the protein begins to adopt increasingly extended structures. For higher charge states between [1]^11+^ to [1]^17+^, extended structures dominate (^TIMS^CCS_N2_ 3000 – 4000 Å). Mobility peaks in this region become sharper and better defined, indicating reduced conformational heterogeneity. This trend agrees with earlier reports showing broader CCS and arrival time distributions for lower charge states and progressively narrower distributions at higher charge states.^49,65^

To examine whether low-flow SEC-MS can separate early stage oligomers of α-syn, we analyzed an aggregated sample using this platform. Figure 2A shows the total ion current (TIC) of the SEC-MS analysis of the aggregated sample. Compared to the fresh α-syn, the retention time shifts to 8.5 min, and the peak becomes significantly broader, indicating the presence of higher-order species. Figure 2B displays the SEC-MS trace, where the monomer is detected predominantly in its folded form, and the unfolded monomer is no longer present. In addition to the monomer, the dimer species is clearly observed. Figure 2C zooms in on the *m*/*z* region between 2700 and 5000 to clearly visualize the higher-order oligomeric species. To obtain a clearer overview of the oligomer distribution, the spectrum was subsequently deconvoluted (Figure 2D), revealing additional early stage oligomers ranging from trimers to hexamers. However, no separation of these oligomers occurs during SEC. This lack of separation is due to several factors. First, early α-syn oligomers do not behave as stable, uniform globular species, but rather as low-abundance (<1% per oligomer), dynamic, and heterogeneous IDP assemblies with broad conformational distributions and overlapping hydrodynamic volumes. Second, SEC separation dilutes the injected sample over the elution volume, lowering the effective concentration of these weakly populated species and making them difficult to detect as discrete peaks. Finally, aggregation is a continuous process: millions of primary nuclei can form within seconds, and when analyzing a 7-day aggregated sample, secondary nucleation can rapidly amplify fibril populations on a time scale that may be faster than, or comparable to, the SEC separation.^49,66^ As a result, the SEC fails to resolve distinct intermediates and cannot capture an accurate snapshot of the early stage oligomeric distribution.

**Figure 2:**
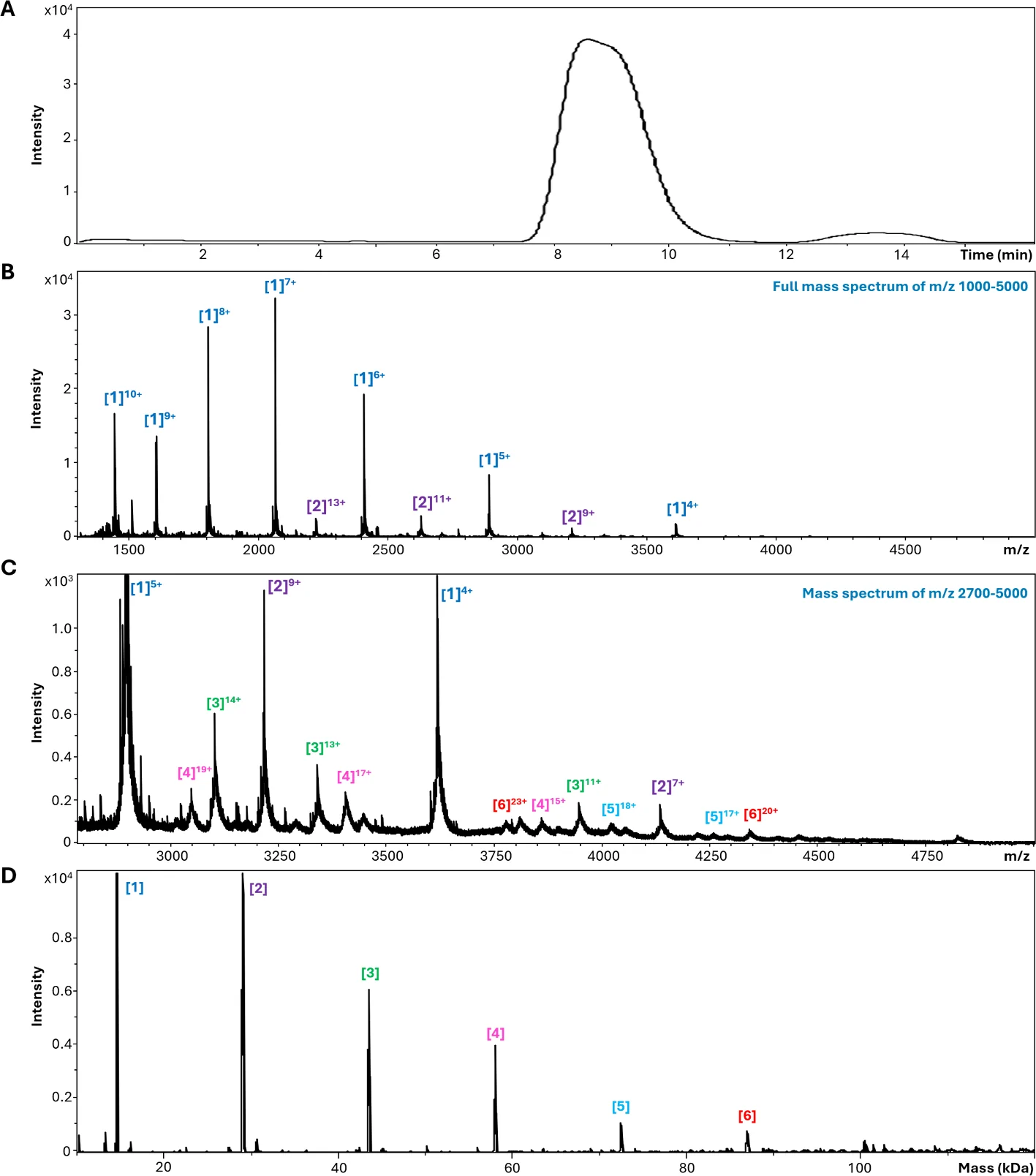
SEC-MS of aggregated α-syn (A) Total ion current of aggregated α-syn in SEC-MS with a flow rate of 16 mL/min resulting in a one broad peak with a retention time of 8.5 min; the second peak at 13.5 min contains only salts. (B) Full mass spectrum of aggregated α-syn between *m* **/** *z* 1000–5000. (C) Mass spectrum of the aggregated α-syn focused on *m*/*z* 2700–5000. (D) Deconvoluted MS of aggregated α-syn where the number of oligomers is assigned on top of the deconvoluted peaks.

### Direct Infusion Nano-ESI Analysis of α-syn Oligomers

Figure 3A shows the direct infusion analysis of a fresh α-syn sample using ESI (top) and online nanoESI (bottom). As can be seen, the fresh α-syn sample exhibits a distinctly different charge state distribution depending on the ionization method used. In direct infusion ESI (Figure 3A, top), we observe a double charge state distribution of the monomer, closely resembling the profile obtained from SEC-MS, with only low levels of dimer species present. α-syn oligomers are very fragile and can dissociate either during the ESI process or during transmission in the mass spectrometer. In contrast, using the online nanoESI source (Figure 3A, bottom), a single broad charge state distribution is observed, ranging from [1]^6+^ to [1]^14+^. This suggests a less extended structure overall, although the most intense peak shifts to [1]^10+^. Notably, the overall MS signal intensity increases by approximately 4-fold with the online nanoESI source. The increased intensity of both the dimer species and the low charge states monomer under these conditions suggests a softer ionization process, likely due to the lower capillary voltage applied (1500 V compared to 4500 V in direct infusion ESI).

**Figure 3:**
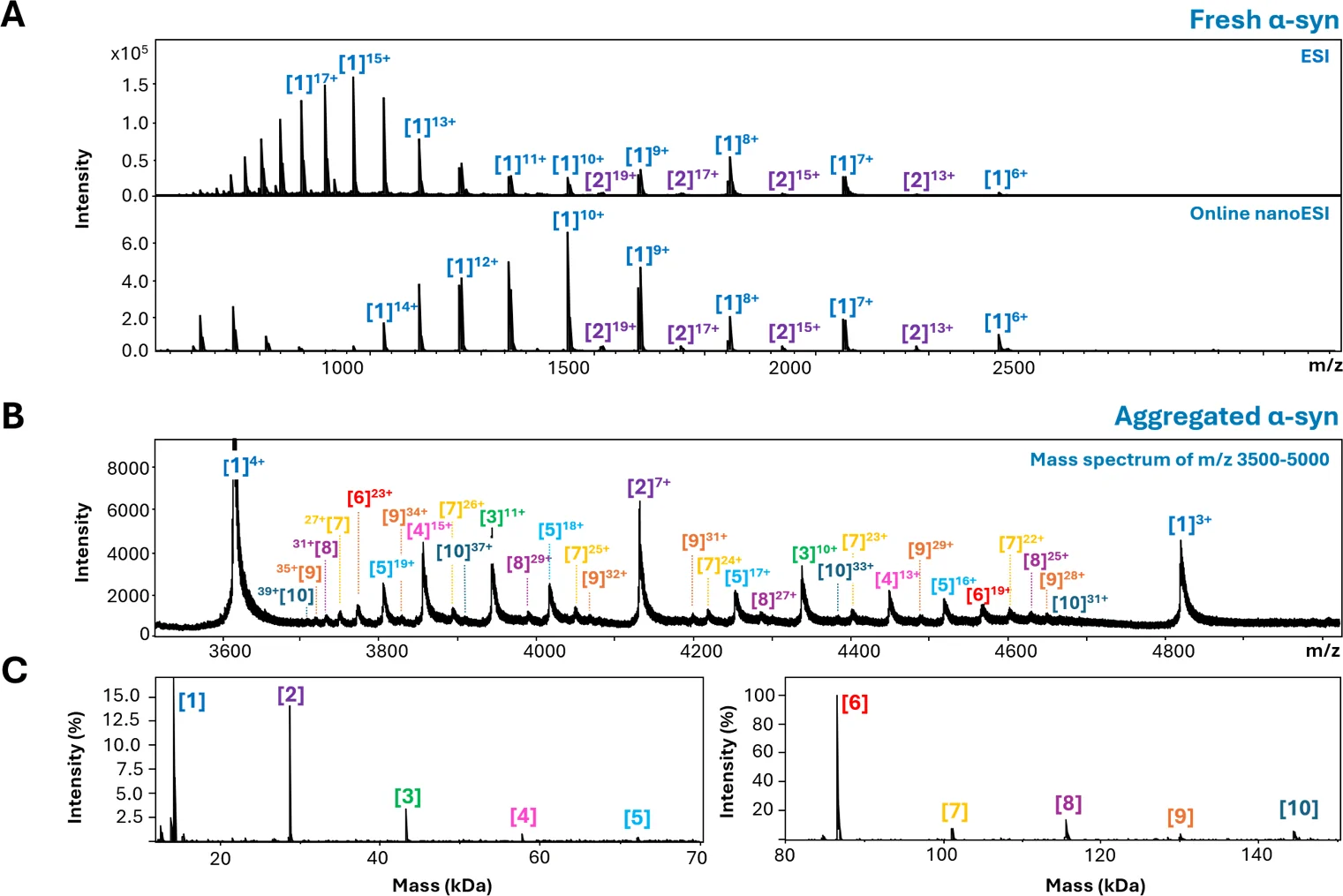
DI-MS of α-syn using ESI and online nanoESI (A) Fresh α-syn sample measured in direct infusion using ESI and online nanoESI source (B) Aggregated α-syn measured with online nanoESI and (C) Deconvolution of the oligomers of α-syn. The left panel (10–70 kDa) displays monomer to pentamer species, while the right panel (80–150 kDa) reveals higher-order oligomers from hexamer to decamer. Intensity scale given as relative abundances as different deconvolution parameters were used for each panel, which can result in inconsistencies in the absolute intensities.

When we applied the direct infusion-nMS to the aggregated α-syn sample, we were able to detect a wide range of oligomeric species. Figure 3B shows the mass spectrum of the aggregated sample, focused on the range of 3500–5000 *m*/*z* to highlight the formation of higher-order oligomers. In this region, we observe charge states corresponding to oligomers up to the decamer. Deconvolution of the mass spectrum (Figure 3C) provides a clearer view of the oligomeric distribution. The left panel displays species ranging from monomer to pentamer, obtained using a deconvolution window of 10–70 kDa. The right panel shows a higher mass range (80–150 kDa), revealing oligomeric species from hexamer to decamer, which have not been previously reported.

### Toward an Optimized TIMS-MS Platform for α-Synuclein Oligomer Analysis

Analyzing the noncovalently bound species such as α-syn oligomers with the TIMS-MS platform is inherently challenging as these species are structurally fragile and prone to dissociation due to collisional heating incurred during ion trapping and mobility separation.^67,68^ Initially, TIMS-MS did not detect any species larger than a dimer, requiring minimized RF and DC voltages, to balance transmission while keeping these delicate protein aggregates intact. Figure 4A shows the TIMS-TOF setup and highlights key parameters used to optimize oligomer transmission and preserve their structure integrity.^69^

**Figure 4:**
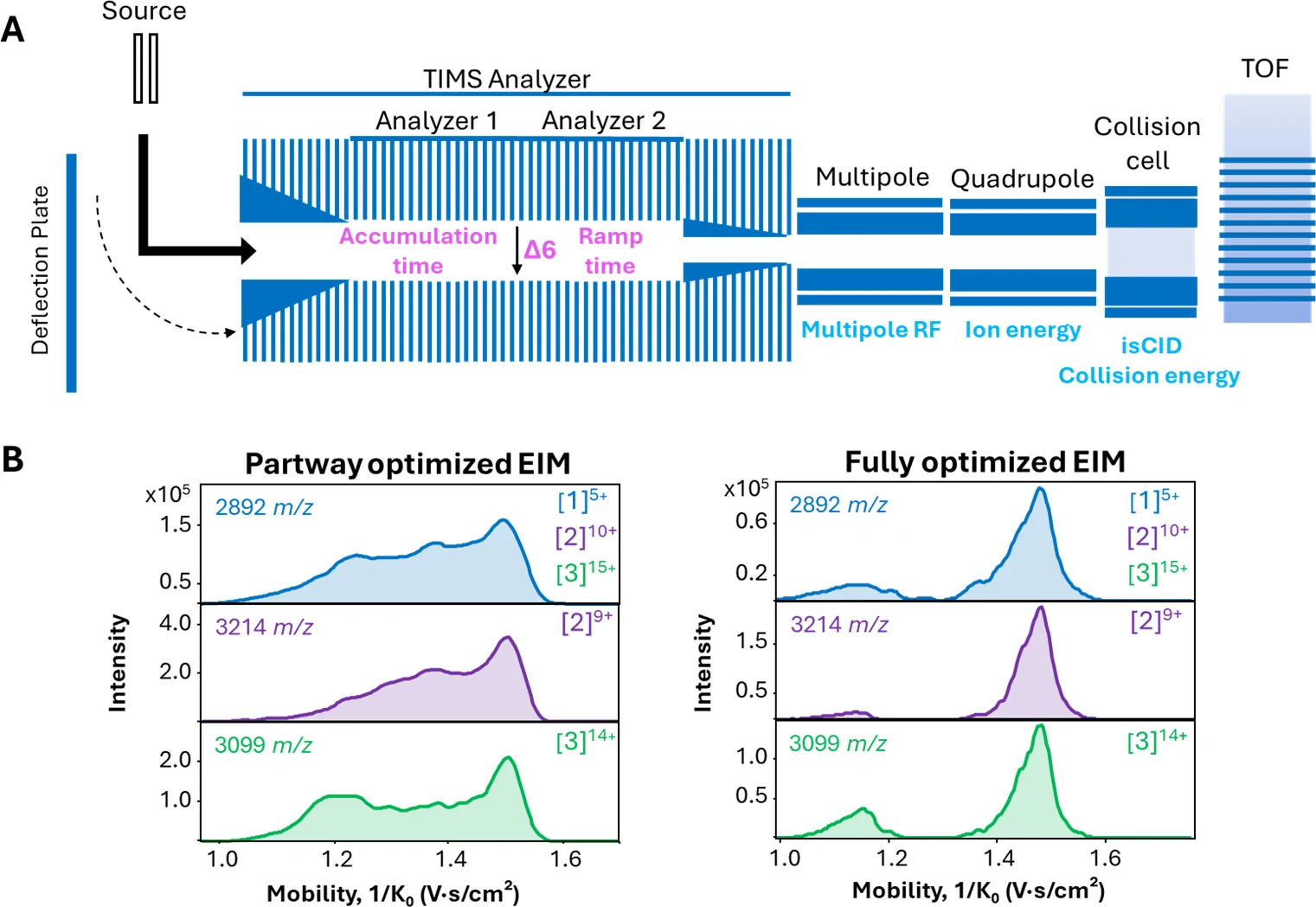
TIMS-TOF optimization for high-order oligomers (A) Schematic of the TIMS-TOF setup with key optimization parameters highlighted. (B) Extracted Ion Mobilogram (EIM) of *m*/*z* 2892, 3214, 3099 corresponding to charge state 5+ of monomer (blue), 9+ of dimer (purple), and 14+ of trimer (green), respectively, before and after full TIMS-TOF optimization.

Within the TIMS funnel (highlighted in pink), the accumulation time, Δ*p*otentials, and ramp time were identified as the most critical parameters governing oligomer stability. Increasing the accumulation time enhances the signal intensity of low-abundance oligomeric species; however, accumulation times above ∼ 50 ms led to pronounced space-charge effects, resulting in distorted mobility profiles, particularly for larger oligomers (Figure S2). To mitigate these effects and avoid undesired ion–ion interactions and delays, the accumulation time was therefore reduced to 20 ms, which provided stable transmission while preserving fragile oligomeric species. The influence of Δ*p*otentials on protein structural integrity is well established,^70–72^ with Δ6 having the most significant effect because it controls the energetic transfer of ions from the TIMS tunnel into the analyzer region and can therefore promote activation, unfolding, or dissociation of fragile protein assemblies.^73^ To minimize collisional activation within the TIMS tunnel, Δ6 was set to 30 V, corresponding to the lowest value permitted by the software for the selected mobility window. Ramp time represents a key parameter in TIMS, as longer ramp times improve mobility resolution but also increase the residence time of ions within the electric field, which can lead to ion heating.^70,74^ For α-syn oligomers, extended ramp times resulted in reduced signal stability and poor signal-to-noise ratios (Figure S3). Consequently, the ramp time was reduced from the standard 100 ms typically used for protein analyses to 50 ms, providing an optimal compromise between separation performance and preservation of oligomeric structures.

Instrumental parameters downstream of the TIMS funnel that govern ion transmission (highlighted in blue in Figure 4A) further contribute to the overall softness of the platform. Multipole RF amplitudes, ion energy, and collision energy were therefore minimized to facilitate efficient transmission of oligomers without inducing fragmentation. In-source CID (isCID) acts as a bias voltage, introducing an energy offset across the ion optics and causing ions to experience a voltage drop at the exit of the TIMS tunnel, particularly at the entrance of funnel 2. While this process can promote declustering and adduct removal, excessive activation risks destabilizing fragile complexes. Interestingly, we found that applying relatively high isCID values (90 V) improved desolvation, without inducing dissociation.

Figure 4B compares the extracted mobilograms obtained during and after full optimization for three representative *m*/*z* values: 2892 *m*/*z*, where the monomer [1]^5+^, dimer [2]^10+^, and trimer [3]^15+^ overlap; 3214 *m*/*z*, corresponding to the dimer [2]^9+^; and 3099 *m*/*z*, corresponding to the trimer [3]^14+^. Without optimization, the mobility spectra are broad and lack resolution, making it difficult to distinguish between compact and extended conformations. After full optimization, the mobility profiles are significantly sharper, with well-defined peaks. This improvement allowed us to better resolve the structural differences between species, particularly in identifying compact and extended forms.

### Soft TIMS-MS Platform for α-Synuclein Oligomer Analysis

The optimized soft nanoESI-TIMS-MS workflow was used to measure aggregated α-syn. Figure 5A presents the TIMS mobilograms as ^TIMS^CCS_N2_ profiles, with the normalized intensity plotted against ^TIMS^CCS_N2_ of α-syn oligomers ranging from dimers to pentamers, assigned according to theoretical masses and charge state series (Table S3). The oligomers are grouped by size and color-coded as follows: dimers (purple), trimers (green), tetramers (pink), and pentamers (light-blue). Each trace corresponds to a distinct charge state which is indicated on the right of each ^TIMS^CCS_N2_ profile. Only charge states unique to each oligomer are plotted; charge states that overlap with monomer or other smaller oligomers have been excluded.

**Figure 5:**
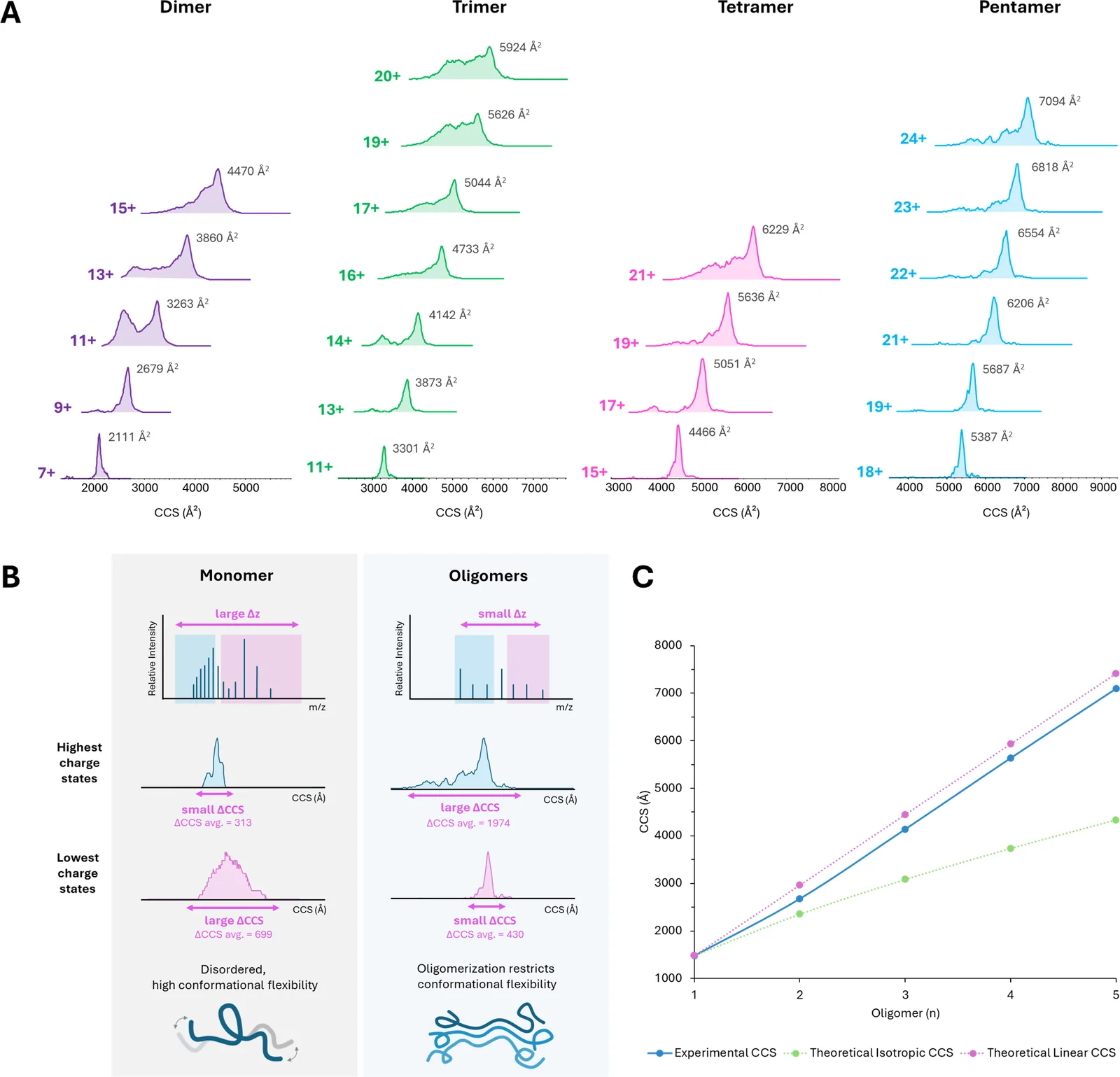
^TIMS^CCS_N2_ landscape of oligomers. (A) ^TIMS^CCS_N2_ of dimer (purple), trimer (green), tetramer (pink), and pentamer (light blue) (B) Schematic representation of the CCS ranges (ΔCCS) corresponding to the lowest and highest observed charge states for monomeric and oligomeric α-synuclein. (C) Experimental ^TIMS^CCS_N2_ values are plotted for an average charge per monomer 5+ for all oligomers and compared to theoretical CCS trends calculated for linear and isotropic growth models.

Examination of the individual oligomers in Figure 5A reveals a clear charge-dependent trend. For all oligomers, higher charge states exhibit broader and less defined mobility features, whereas lower charge states display sharper and more distinct peaks. In addition, as the charge decreases, the contribution of a compact conformational family vanishes, as indicated by the disappearance of an extra shoulder peak at low ^TIMS^CCS_N2_ values. This behavior is consistent across all oligomers but contrasts with that of the monomer. The monomer showed the opposite trend; sharper features at higher charge states and broader distributions at lower charge states (see Figure 1C in Section 3.1).

The observed charge-state-dependent mobility behavior is a result of fundamental differences in the solution-phase structures between monomeric and oligomeric α-syn. Monomeric α-syn is known to exist in solution as a highly heterogeneous ensemble of rapidly interconverting conformations. These conformations are transferred to the gas phase via several ionization mechanisms, with their charge states reflecting their solvent accessible surface area (SASA) from solution. Compact monomer conformations are preferentially ionized via the charge residue model (CRM), in which the protein remains embedded within the evaporating droplet until solvent removal is complete, resulting in ions that carry relatively few charges.^75^ Extended monomer conformations follow the chain ejection model (CEM), where elongated protein chains are exposed from the droplet surface and are progressively expelled, accumulating a larger number of charges in the process.^65,75^ For partially compact monomer conformations, Beveridge et al. propose an intermediate mechanism between the CRM and CEM whereby the extended region of the protein is first ejected via the CEM, while the folded region will remain inside the droplet and are ionized according the CRM, resulting in intermediate charge states.^75,76^ Once in the gas phase, the extended conformations with high charge states can experience strong Coulombic repulsion between the charges which restricts conformational rearrangement, confining these ions to a narrower set of extended ^TIMS^CCS_N2_ conformations.^77,78^ This can explain why we observe sharper, better-defined mobility features at higher charge states (Charge states [1]^11+^ to [1]^17+^ in Figure 1C Section 3.1). In contrast, folded conformations with lower charge states experience weaker Coulombic repulsion, allowing greater structural flexibility in the gas phase. This can permit interconversion between multiple compact conformations during the experiment. When these transitions occur on time scales comparable to or faster than ion mobility separation, the resulting mobility distributions appear broader and less defined than those of the more extended states (charge states [1]^6+^ and [1]^7+^ in Figure 1C, Section 3.1).^77^ Partially folded conformations retain both compact and extended states, producing a broad ^TIMS^CCS_N2_ distribution spanning the folded–unfolded conformational landscape (Charge states [1]^8+^ and [1]^9+^ in Figure 1C, Section 3.1).

For oligomeric α-syn, this relationship changes. Oligomerization appears to partially constrain the disordered monomer units through intermolecular contacts, reducing conformational freedom. As a consequence, oligomers exhibit narrower charge-state distributions, sharper mobility peaks at lower charge states, and occupy less of the conformational space, indicating increased structural stabilization. At higher charge states, oligomeric α-syn shows broader ^TIMS^CCS_N2_ features, in contrast to the monomer. This likely reflects greater structural diversity among higher-charge oligomers, where more extended and flexible monomer subunits contribute to a wider range of possible oligomer arrangements. In contrast, lower-charge oligomers may arise from more compact subunits with reduced solvent-accessible surface area and more restricted conformational freedom. These charge-state-dependent trends suggest that the observed gas-phase structures retain information about solution-phase conformational diversity. Overall, however, oligomerization leads to narrowing of both ΔCSD and Δ ^TIMS^CCS_N2_ indicating reduced flexibility and increased structural rigidity as α-syn progresses from monomer to higher-order oligomers.

The differences we observe are consistent with previous molecular dynamic simulations showing that dimerization of α-syn increases β-sheet content and reduces the conformational disorder characteristic of the monomer.^79^ Similarly, simulations of the intrinsically disordered protein amyloid-β have demonstrated that the monomer preferentially occupies a low-energy disordered ensemble, whereas for the dimer, the lower energy state is more ordered, resembling folded conformations.^80^ Overall, our observed differences in charge-state and conformational distributions between monomeric and oligomeric species align with prior reports that oligomerization is accompanied by increased structural ordering relative to the monomer.

Comparing the different oligomers provides further insight into the early stage of the aggregation mechanism. When examining the effect of increasing the number of monomer units (n) at a constant charge state (z), the mobility features become progressively narrower with increasing oligomer size. For instance, at charge state 19+, the mobility distribution evolves from broader and less defined features for the trimer (green) to markedly sharper and more well-defined peaks for the pentamer (light blue) (Figure 5A). Despite the increase in oligomer size, the ^TIMS^CCS_N2_ values remain relatively constant across the series (trimer ^TIMS^CCS_N2_ = 5642 Å^2^, tetramer ^TIMS^CCS_N2_ = 5635 Å^2^, pentamer ^TIMS^CCS_N2_ = 5677 Å^2^), suggesting that oligomer growth occurs without proportional expansion of the overall cross section. This trend may reflect two contributing effects. First, as oligomer size increases at fixed charge, the charge per monomer unit decreases (z/n = 6.3, 4.8, and 3.8 for trimer, tetramer, and pentamer, respectively), reducing intramolecular Coulombic repulsion and thereby favoring more compact conformations in the gas phase. Second, oligomer growth can be accompanied by structural reorganization toward more ordered and stabilized assemblies, which would reduce conformational heterogeneity and thus yield narrower, more well-defined ion mobility features. Figure 5B presents a schematic representation of the CCS ranges corresponding to the lowest and highest charge states observed for monomeric and oligomeric α-syn.

To better understand oligomer growth, it is necessary to minimize the effects of charge-induced expansion by comparing charge states that yield a similar charge per monomer unit across different oligomers. In this case charge state 5+ for monomer, 9+ (z/n = 4.5) for dimer, 14+ (z/n = 4.7) for trimer, 19+ (z/n = 4.8) for tetramer, and 24+ (z/n = 4.8) for pentamer were selected. The experimental ^TIMS^CCS_N2_ values of these charge states were plotted (blue) and compared to the theoretical isotropic (green) and linear (pink) growth curves based on the monomer (Figure 5C) according to the Bleiholder model.^81^ Further details for experimental values and growth calculations can be found in Table S2 and Figure S4 of the Supporting Information. Figure 5C illustrates that the ^TIMS^CCS_N2_ values follow closely a linear trend rather than isotropic growth. This linear growth reflects a structured β-sheet stacking growth, in contrast to the unstructured spherical growth predicted by isotropic growth.^81,82^ A similar behavior was reported by Khaled et al. for amyloid-β, where linear growth was observed for assemblies larger than *n* > 4.^82^

The linear β-strand type growth is further supported by comparing our mobility data to the structural models illustrated for oligomers by Bleiholder et al. They propose that for the same aggregation state, i.e. same number of monomer units (n), compact structures with lower CCS are associated with globular conformations, whereas larger CCS values are assigned to a more extended β-strand-like architecture.^81^ Although their proposed process is based on information derived from helium-based drift tube measurements, the trends observed remain informative for our TIMS experiments in N_2_. In our TIMS data, dimers and trimers clearly display a compact conformational family at lower ^TIMS^CCS_N2_ values, while for tetramers and pentamers, these compact structures are largely lost (Figure 5A). This further illustrates a progressive loss of compact globular structures and a shift toward the extended structures as oligomers increase in size. This indicates an on-pathway fibrillar growth trend for α-syn, matching the trend exhibited by the growth curve.

### From Functional Flexibility to Pathological Rigidity of α-Synuclein

In the healthy brain, α-syn regulates neurotransmission at the presynaptic terminal by modulating synaptic vesicle clustering, docking, and fusion.^6^ Rather than acting as a rigid structural protein, it functions as a flexible regulator whose disordered structure is central to its role. Upon binding curved or anionic lipid membranes, the N-terminal region adopts amphipathic α-helices that transiently anchor the protein to vesicles and facilitate SNARE (the machinery that physically drives synaptic vesicle-membrane fusion) complex assembly.^83^ The C-terminal region remains largely disordered, buffering electrostatic interactions and suppressing irreversible aggregation. Through this reversible folding behavior, α-syn maintains synaptic balance and plasticity.^84^ This functional plasticity is directly reflected in the conformational behavior of monomeric α-syn observed in our TIMS measurements. The data shows that α-syn samples a wide structural landscape, ranging from compact and heterogeneous low-charge ensembles to more extended and conformationally restricted higher-charge states. This structural flexibility is consistent with the physiological role of α-syn as a dynamic protein that can reversibly engage membranes and contribute to synaptic vesicle regulation. Importantly, upon extended incubation (7 days and following SEC separation), the bimodal CSD in which both a folded and unfolded charge state envelope are present (Figure 1B), characteristic of fresh α-syn sample, disappears. Thereafter, only the lower charge state envelope is retained, indicating a loss of the conformationally extended monomer population (Figure 2B). This indicates that the population of freely fluctuating monomers decreases over time as α-syn transitions toward more stable assemblies.

The same structural plasticity that underlies the normal function of α-syn also predisposes the protein to self-association into metastable oligomers. During oligomerization, α-syn shifts from a highly dynamic monomeric ensemble, capable of transient folding upon membrane contact, toward more structurally persistent assemblies, which have been reported in previous studies to acquire increased ordered or β-sheet-rich character.^85^ While TIMS does not directly report on secondary structure, our data show that upon α-syn oligomerization from dimers to pentamers (Figure 5B), the broad conformational heterogeneity characteristic of early species progressively decreases, with reduced CSDs and mobility profiles that become sharper and more defined. This indicates a reduction in gas-phase conformational diversity and an increase in structural stabilization within the detected oligomeric ensemble. Notably, the reduced relative abundance of compact conformational families as oligomerization progresses, can suggest that oligomerization selectively decreases the population of flexible, monomer-like states, resembling β-strand-like linear growth.^80^

Although we do not directly measure membrane activity in the presented work, our results support a proposed structural model in which oligomer formation reduces the conformational flexibility of α-syn. Previous studies have shown that α-syn oligomers can interact with lipid membranes, insert into bilayers, permeabilize model membranes, and disrupt cellular membranes.^86,87^ Therefore, the increased rigidity observed in our oligomeric species may indicate the shift of α-syn away from finely tuned, reversible membrane engagement and toward more aberrant membrane interactions. Functionally, this marks a transition from a dynamic, regulatory protein to a structurally constrained, membrane-disruptive species.^6,83^ Our TIMS data, therefore, provide direct experimental support for the concept that early α-syn oligomerization represents a critical tipping point at which physiological disorder-based function is replaced by pathological rigidity.

## Conclusion

In this work, we establish a gentle, multimodal mass spectrometry platform that resolves α-synuclein across its early aggregation landscape and probes changes in conformational dynamics during oligomer formation. Recognizing that both SEC and TIMS are underutilized for intrinsically disordered proteins and their early stage oligomers, we evaluated their respective capabilities, both individually and in tandem, across both monomeric and oligomeric states while preserving fragile assemblies. Low-flow SEC-TIMS-MS allows detection of broad ensemble-level differences between fresh and aggregated α-syn, rather than the complete resolution of the underlying oligomeric conformational states. While online nanoESI with soft TIMS conditions can preserve fragile oligomers, enabling mass detection up to the decamer level and structural characterization up to the pentamer.

Our measurements reveal a clear structural progression during early oligomerization: conformational heterogeneity decreases while structural stabilization increases from dimers to pentamers. Whereas monomeric α-syn populates a broad dynamic ensemble consistent with its known structural plasticity, oligomer formation progressively restricts this conformational flexibility. The data support a model in which α-syn undergoes a near-linear structural progression from a highly dynamic monomeric ensemble toward increasingly persistent and rigid oligomeric assemblies. While TIMS does not directly identify secondary structure, membrane activity, or toxicity, this loss of conformational heterogeneity may be relevant to previously proposed mechanisms in which α-syn oligomerization alters reversible membrane engagement and promotes aberrant membrane-associated behavior. More broadly, this work demonstrates that native TIMS-MS can directly resolve structural transitions in early stage transient protein assemblies that have largely remained experimentally inaccessible. By identifying how conformational heterogeneity changes during early α-syn oligomerization, these findings define a critical window in early α-syn aggregation that may be particularly relevant for therapeutic intervention in Parkinson’s disease.

## Supplementary Material



## Data Availability

The data underlying this study are openly available in DataCite Commons at 10.48338/VU01-QKG39E.

## Notes

Overview of all TIMS parameters used for α-syn experiments; Raw ion mobility analysis of monomeric α-syn by SEC-TIMS-MS; Direct infusion of α-syn at different accumulation times; Direct infusion of α-syn at different ramp times; Theoretically calculated collision cross-section values for α-syn oligomers; Experimental collision cross-section values for α-syn oligomers (PDF)
