# Supplementary material for: Hyphenated Mass Spectrometry-Based Strategies for Characterizing Oligomers of α‑Synuclein in Parkinson’s Disease
Source: ACS Cent Sci. 2026 Aug 20;12(9):1360–71. doi: 10.1021/acscentsci.6c00514 (PMC13614029; doi:10.1021/acscentsci.6c00514)
Supplement: Supplementary file 1 [file oc6c00514_si_001.pdf]

Supplementary information

Hyphenated Mass Spectrometry-based Strategies for Characterizing Oligomers of α-Synuclein in Parkinson's Disease

Raya Sadighi[1,2], Andrea Istrati[1,2], Sigourney Karijodikoro[1], Guus Scheefhals[3], Melissa Baerenfaenger[1,2], Anouk M. Rijs[1,2]

1. Division of BioAnalytical Chemistry, Department of Chemistry and Pharmaceutical Sciences, Amsterdam Institute of Molecular and Life Sciences, Vrije Universiteit Amsterdam, De Boelelaan 1108, 1081 HZ Amsterdam, The Netherlands
2. Centre for Analytical Sciences Amsterdam (CASA), The Netherlands
3. Syngle Therapeutics B.V., The Netherlands

Corresponding Author: a.m.rijs@vu.nl

Table of Contents

Item	Description	Page
**Section S1**	**Materials and Methods**	**S2**
S1.1	Reagents, chemicals, and α-syn sample preparation	S2
S1.2	SEC-UV and SEC-MS analysis	S2
S1.3	Native ion-mobility mass spectrometry	S2
Table S1	Overview of MS and TIMS acquisition parameters for monomeric and aggregated α-syn	S3
S1.4	Data analysis, CCS determination, and deconvolution	S4
Figure S1	SEC-TIMS-MS analysis of monomeric α-syn and isotopic assignment of monomeric species	S4
Figure S2	Effect of TIMS accumulation time on direct-infusion α-syn analysis	S5
Figure S3	Optimization of TIMS ramp time for nanoESI-TIMS-MS analysis of α-syn oligomers	S6
Table S2	Experimental and theoretical CCS values used for oligomer growth-curve analysis	S6
Figure S4	Comparison of experimental CCS values with isotropic and linear growth models	S7
Table S3	Overview of theoretical and experimentally derived masses for α-syn monomer and oligomers	S7

Materials and Methods

S1.1 Reagents and chemicals

Ammonium acetate (AmAc), sodium phosphate monobasic monohydrate, sodium phosphate dibasic (anhydrous), bovine serum albumin, cytochrome-C, uracil, α-Cyano-4-hydroxycinnamic acid, hydrochloric acid and sodium hydroxide were all obtained from Sigma-Aldrich. Ethanol absolute (dehydrated), acetonitrile, trifluoroacetic acid and formic acid were purchased from Biosolve. Ultrapure water was obtained with a Milli-Q Purification system (Merck Millipore). Human recombinant and aggregated α-syn were provided by Syngle Therapeutics (Utrecht, the Netherlands). Lyophilized human recombinant α-syn was suspended in 50 mM AmAc with a pH of 6.8 to a final concentration of 1 mg/mL and stored at -20°C. Aggregated α-syn was prepared via incubation at room temperature for 7 days.

S1.2 SEC analysis

SEC-UV and SEC-MS measurements were carried out on an UltiMate 3000 UHPLC platform (Thermo Fisher Scientific) equipped with an UltiMate WPS-3000TFC analytical autosampler, an UltiMate NCS-3500RS nano pump, and an UltiMate VWD-3400RS UV detector. Separations were performed using a TSKgel SuperSW3000 SEC column (1.0 mm I.D. × 30 cm, 4 µm; TOSOH Bioscience). The mobile phase consisted of 150 mM ammonium acetate at pH 6.8 delivered at 16 µL/min. Samples (1 µL) were injected and monitored by UV absorbance at 280 nm.

S1.3 Native Ion-Mobility Mass Spectrometry

A timsTOF Pro 2 instrument (TIMS-Qq-TOF, Bruker) was used in positive-ion mode for all experiments. Monomeric α-syn was characterized using both SEC-ESI-TIMS and direct-infusion nanoESI–TIMS workflows, the latter performed with the CaptiveSpray 2 ion source (Bruker), henceforth called "dynamic nESI" to distinguish from static nESI, at a tunnel-in pressure of 2.5 mbar. Aggregated samples were analyzed using a softened nanoESI–TIMS method at 2.7 mbar. Samples were introduced by flow injection, using a 1 µL sample injection and a 1 µL/min flow of 50 mM AmAc. The instrument was calibrated for both mass and mobility using Agilent ESI tune mix under the respective tunnel-in pressure conditions prior to analysis. An overview of all acquisition parameters is provided in Table S1.

Table S1. Overview of all MS and TIMS parameters used for characterization of monomer and aggregated α-syn.

Parameter	SEC-TIMS Monomer	nESI-TIMS Monomer	nESI-TIMS aggregate
Capillary voltage (V)	4500	1500	1500
Dry gas (L/min)	10	10	8
Dry temperature (°C)	200	200	180
Deflection delta (V)	70	70	70
Funnel 1 RF (Vpp)	300	300	350
Funnel 2 RF (Vpp)	600	600	600
isCID (eV)	40	40	90
Ion energy (eV)	5	5	2
Collision energy (eV)	10	10	4
Transfer time (µs)	90	90	130
Multipole RF (Vpp)	500	500	200
Collision RF (Vpp)	3200	3200	3500
Prepulse Storage (µs)	20	20	20
Δ1 (V)	50	50	-50
Δ2 (V)	-150	-150	-150
Δ3 (V)	70	70	70
Δ4 (V)	100	100	100
Δ5 (V)	0	0	0
Δ6 (V)	150	150	30
Ramp time ($V \cdot s/cm^2$)	70	70	50
Accumulation time (ms)	50	50	20
$1/K_0$	0.9 – 2.0	0.9-2.0	0.8 – 1.6
Tunnel in vacuum (mBar)	2.5	2.5	2.7

S1.4 Data analysis

UV chromatograms, mass spectra and extracted ion mobilograms were obtained and analyzed using Bruker Compass DataAnalysis (V6.1). After assigning charge states to the extracted ion mobilograms, experimental $^{TIMS}CCS_{N2}$ distributions were determined for each species. The $^{TIMS}CCS_{N2}$ values of each oligomer were subsequently plotted against theoretical isotropic and linear growth models. The theoretical growth curves were calculated based on the experimentally measured monomer $^{TIMS}CCS_{N2}$ obtained in the same run as the oligomers, following the equations described by Bleiholder *et al.*[60] To isolate size-dependent growth effects, experimental $^{TIMS}CCS_{N2}$ values were charge-normalized according to oligomeric state to minimize charge-related contributions. Further detail on the oligomer growth plots can be found in Table 5 of the Supporting Information. Spectra were deconvoluted using the MaxEnt (maximum entropy) algorithm implemented in Bruker Compass DataAnalysis. For aggregated α-syn, deconvolution was performed in two distinct mass windows (10-70 kDa and 70-150 kDa) to accurately resolve lower- and higher-order oligomeric species.

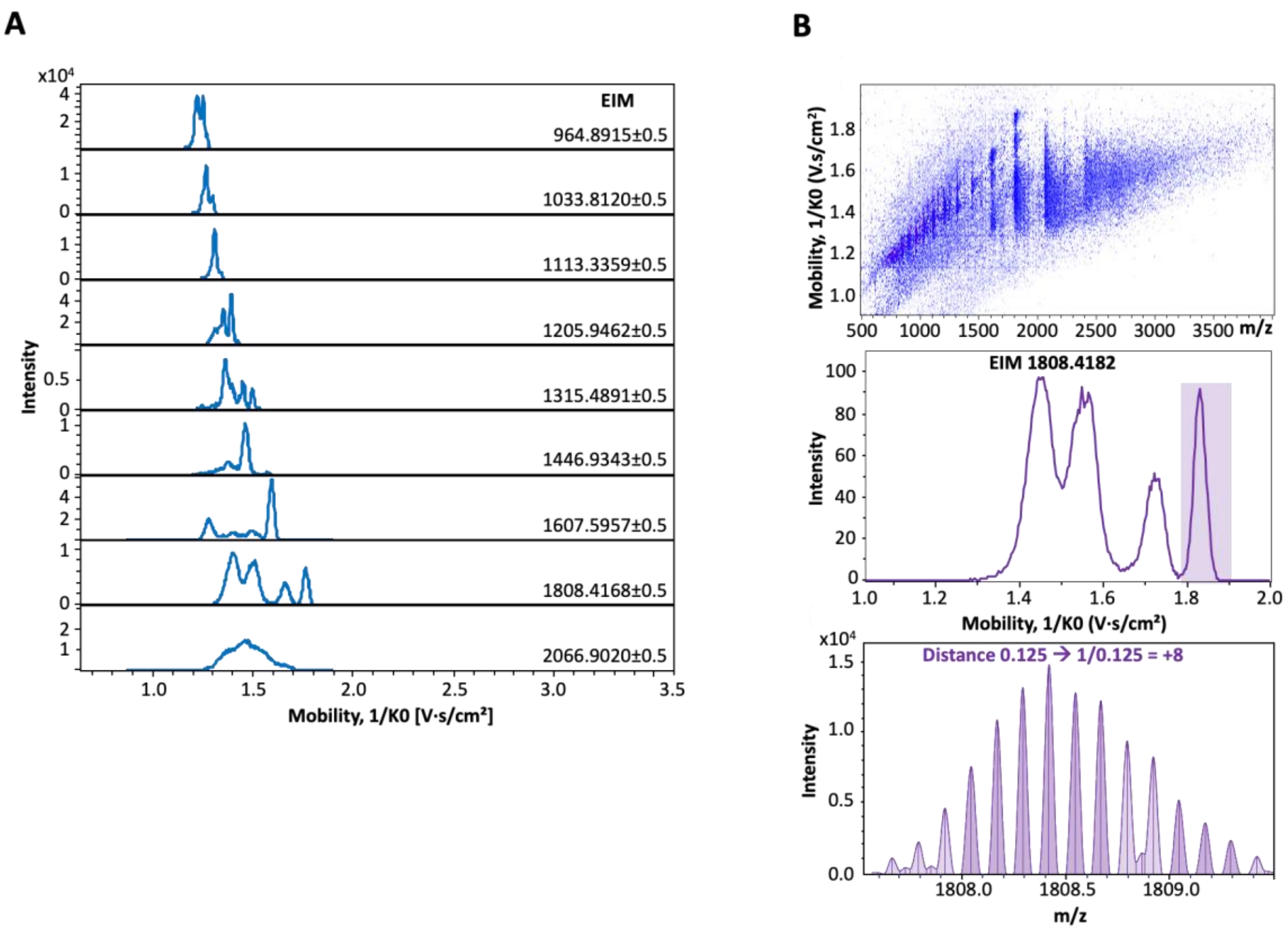

Figure S1. Raw ion mobility analysis of monomeric α-syn obtained by SEC-TIMS-MS. **(A)** Experimental inverse reduced mobility ($1/K_0$) values measured for charge states $[M+7H]^{7+}$ to $[M+15H]^{15+}$ of α-syn monomer. **(B)** Two-dimensional heat map of α-syn showing $1/K_0$ plotted as a function of *m/z*. At *m/z* 1808, corresponding to the $[M+8H]^{8+}$ charge state, extraction of the mobility-resolved mass spectrum and inspection of the isotopic distribution reveal exclusively monomeric α-syn, with no detectable dimer contribution. This suggests that under the applied SEC-TIMS conditions, dimeric species are not preserved, indicating that the method is not sufficiently gentle to maintain these non-covalent assemblies and therefore predominantly detects monomeric α-syn.

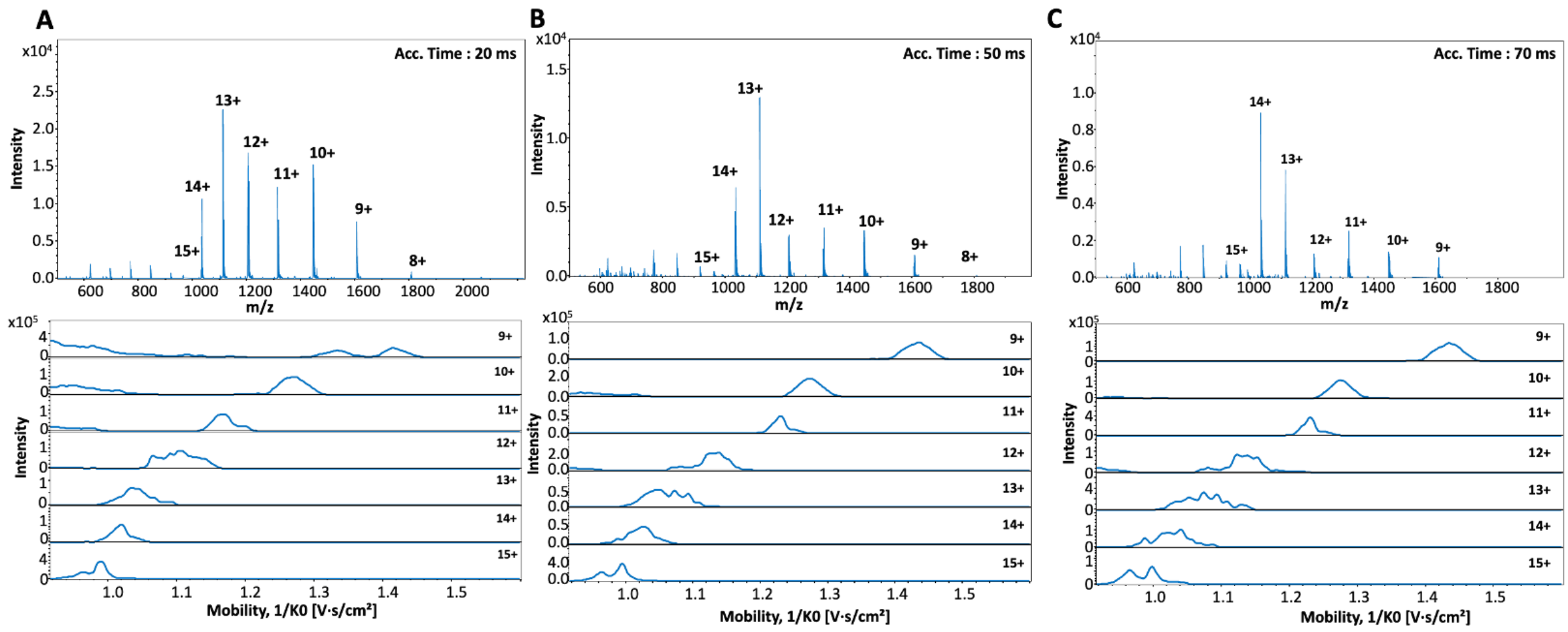

Figure S2. Direct infusion of α-syn analyzed by TIMS-MS at increasing accumulation times. Mass spectra obtained at accumulation times of **(A)** 20 ms, **(B)** 50 ms, and **(C)** 70 ms. At 20 ms accumulation time, the charge state distribution is shifted toward lower charge states, consistent with more compact conformations of α-syn in the gas phase. However, due to the short ramp time (20 ms), the apparent low mobility feature is an ion-rollover effect. As the accumulation time increases to 50 ms and further to 70 ms, the charge state distribution progressively shifts toward higher charge states (at a ramp time of 50 ms). This shift indicates the presence of more extended and unfolded conformations. The observed transition suggests that longer accumulation times lead to increased ion density in the accumulation region, promoting space-charge effects and enhanced ion–ion interactions. These conditions can induce partial gas-phase unfolding, resulting in higher charge states and a broader conformational distribution. Therefore, 20 ms was selected as optimal.

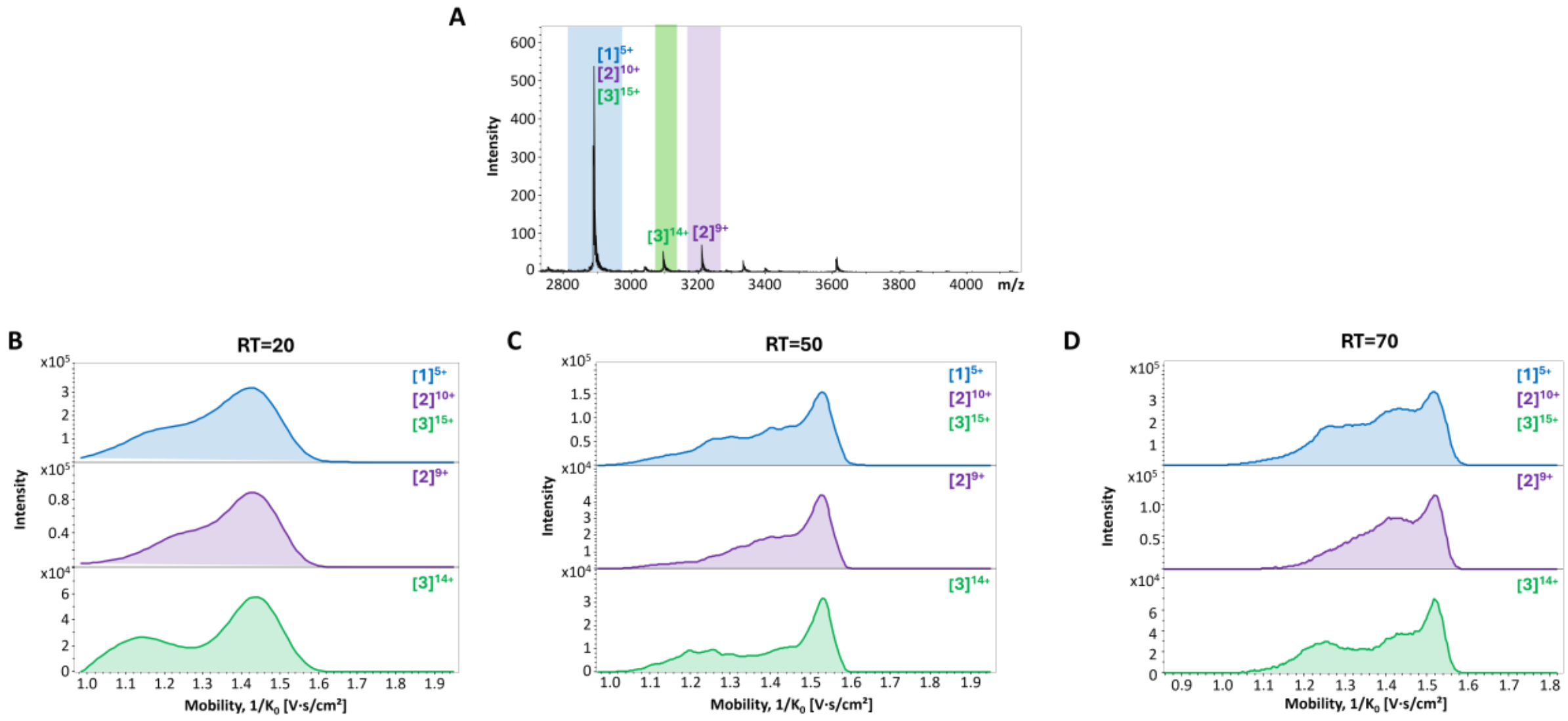

Figure S3. Optimization of TIMS ramp time for nanoESI-TIMS-MS analysis of α-syn oligomers.

(A) Representative mobility spectra of α-syn oligomers highlighting three selected *m/z* values. Mobility distributions obtained at ramp times of **(B)** 20 ms, **(C)** 50 ms, and **(D)** 70 ms. At a ramp time of 20 ms, the separation is insufficient for ions to reach steady-state trapping conditions, resulting in premature elution and reduced mobility resolution. In contrast, at a ramp time of 70 ms, ions become increasingly unstable within the TIMS tunnel, leading to signal deterioration and partial dissociation of oligomeric species. A ramp time of 50 ms was chosen for effective mobility separation while preserving the integrity of α-syn oligomers

Table S2. Overview of experimental and theoretically calculated collision cross section (CCS) values used for growth curve analysis.

Oligomer	m/z	z	z/n	Experimental CCS	Theoretical Isotropic CCS	Theoretical Linear CCS
1	2892.9	5	5	1482.6	1482.6	1482.6
2	3214.3	9	4.5	2679.2	2353.5	2965.2
3	3099.5	14	4.7	4141.5	3083.9	4447.8
4	3045.0	19	4.8	5635.9	3735.9	5930.4
5	3014.7	24	4.8	7093.8	4335.1	7413.0

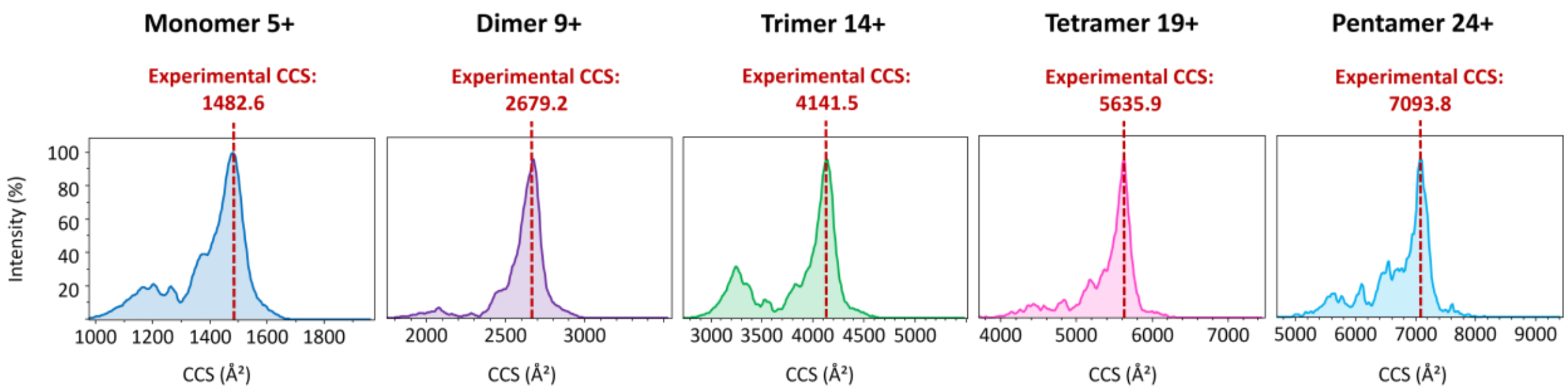

Figure S4. Overview of experimental and theoretically calculated collision cross section (CCS) values used for growth curve analysis. Experimental CCS values were determined as the midpoint of the most abundant peak in the $^{TIMS}CCS_{N2}$ profiles for monomer and all oligomers at a normalized charge state of 5+. Theoretical CCS values were calculated assuming isotropic growth ($\sigma_n = \sigma_1 \cdot n^{2/3}$) and linear growth ($\sigma_n = \sigma_1 \cdot n$), following the model described by Bleiholder *et al*.

Table S3. Overview of theoretical and experimentally derived masses for α-syn monomer and oligomers.

α-syn species	Theoretical mass (MW)	Experimental derived (MW)	Mass accuracy (ppm)
Monomer	14460.16	14460.27	7.6 ppm
Dimer	28920.32	28920.46	4.8 ppm
Trimer	43380.48	43380.65	3.92 ppm
Tetramer	57840.64	57841.55	15.7 ppm
Pentamer	72300.80	72299.50	-17.9 ppm
